# Acute 40% exchange-transfusion with hemoglobin-vesicles in a mouse pneumonectomy model

**DOI:** 10.1371/journal.pone.0178724

**Published:** 2017-06-16

**Authors:** Mitsutomo Kohno, Tatsuhiko Ikeda, Ryo Hashimoto, Yotaro Izumi, Masazumi Watanabe, Hirohisa Horinouchi, Hiromi Sakai, Koichi Kobayashi, Masayuki Iwazaki

**Affiliations:** 1 Division of General Thoracic Surgery, Department of Surgery, Tokai University School of Medicine, Isehara, Kanagawa, Japan; 2 Department of Surgery, Keio University School of Medicine, Tokyo, Japan; 3 Department of General Thoracic Surgery, Saitama Medical Center, Saitama Medical University, Kawagoe, Saitama, Japan; 4 Department of General Thoracic Surgery, Saitama City Hospital, Saitama, Japan; 5 Department of Chemistry, School of Medicine, Nara Medical University, Kashihara, Nara, Japan; University of Nottingham, UNITED KINGDOM

## Abstract

**Objectives:**

Hemoglobin vesicles (HbVs) function as a red blood cell (RBC) substitute and are composed of purified hemoglobin encapsulated in a phospholipid bilayer membrane. The performance of HbVs as a substitute for RBC transfusions was examined in a mouse model of pneumonectomy following acute 40% exchange-transfusion with HbVs.

**Methods:**

Before performing left pneumonectomies, 40% of the blood volume of mice was replaced with a) lactated Ringer’s solution (control), b) 5% recombinant human serum albumin (rHSA), c) mouse RBCs shed in rHSA (mRBCs/rHSA), or d) HbV suspended in rHSA (HbV/rHSA). We compared postoperative a) survival, b) functional recovery, and c) histopathological, immunohistochemical, and inflammatory responses among the study groups.

**Results:**

In the HbV/rHSA and mRBC/rHSA groups, all mice survived ≥7 days after pneumonectomy, whereas 100% of the control mice died within a few h and 50% of mice in the rHSA group died within 24 h after pneumonectomy. Immunohistochemical staining for hypoxia-inducible factor-1α showed that hepatic and renal hypoxic injuries were prominently mitigated by HbV and mRBCs.

**Conclusions:**

The oxygen-carrying performance of HbV was similar to that of mRBCs, even with impaired lung functions following pneumonectomy. HbV infusion did not interfere with the recovery from surgical injury. In the near future, HbVs could be used clinically as a substitute for the perioperative transfusion of RBCs, when or where donated RBCs are not immediately available.

## Introduction

In the event of disasters, large amounts of blood are immediately required. However, sufficient blood might not be available, as was the case during the 2011 Tōhoku earthquake in Japan. In addition, there are isolated islands or areas all over the world where donated blood cannot be provided immediately. In some cases, this is because donated blood cannot be stored for long periods. Another problem is that a stable blood-transfusion supply may become difficult to obtain in aging societies, such as in Japan. The Japanese Red Cross Society predicts a blood shortage equivalent to that required for 890,000 people per year in 2027.[1] More effective utilization of donated blood resources will be necessary.

**Fig 1:**
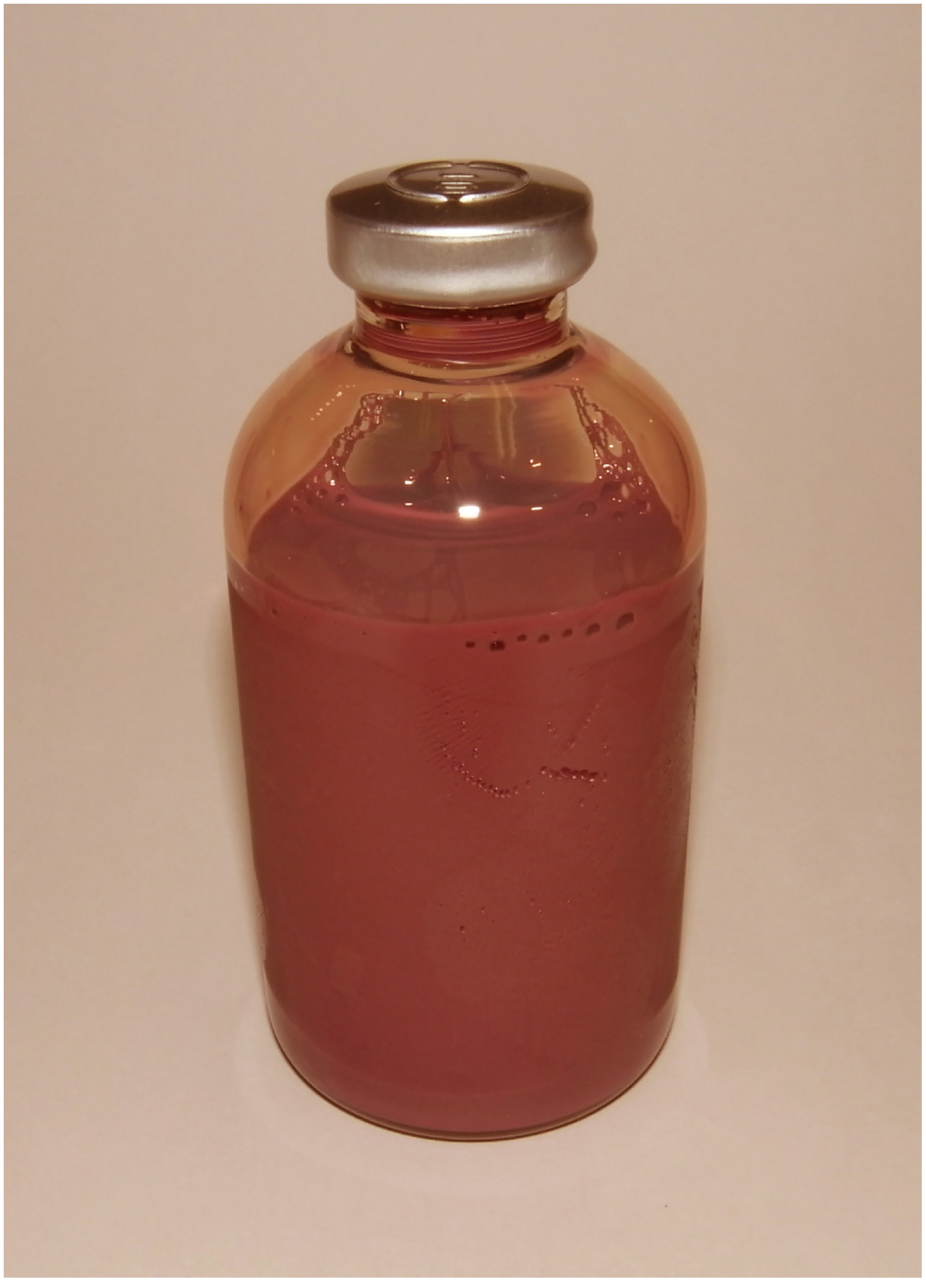
Hemoglobin vesicle (HbV) solution. HbVs were developed as artificial oxygen carriers in the form of liposomes containing concentrated hemoglobin, which can be preserved for years, used regardless of the blood type, and do not pose a risk of infection.

To date, various efforts have been made to develop safe RBC substitutes or artificial oxygen carriers.[2–4] However, no red blood cell substitute has been put into practical use to date. Hemoglobin vesicles (HbVs) have been developed as artificial oxygen carriers in the form of liposomes containing concentrated hemoglobin, which is extracted from outdated human RBCs prepared for transfusion, purified, virus-inactivated, and encapsulated in liposomes (Fig 1).[5] Liposomes have a 250-nm diameter, polyethylene-modified surface with a P_50_ of 28 Torr. They can be preserved for years, used regardless of the blood type, do not pose a risk of infection, and are expected to be clinically applicable in the near future.[6]

HbVs act as oxygen carriers in animal models of 90%-exchange transfusions. HbVs have also been used successfully in experimental resuscitation from hemorrhagic shock. [7–9] Their safety has been evaluated by measuring variations in cytokine concentrations and platelet activation associated with their exposure. Pharmacodynamic, serologic, or metabolic analysis of the reticuloendothelial system has also been performed, [10–12] as have preclinical studies of the long-term survival of dogs after exchange transfusion of HbV.[13] While HbVs have been used to prime the fluid of heart-lung machines,[14] their perioperative effects have not been evaluated. In this study, we examined the performance of HbVs in a mouse model of pneumonectomy by monitoring postoperative survival and recovery.

## Materials and methods

### Animal preparation

Specific-pathogen free, inbred, 8-week-old, male C57BL/6 mice, weighing between 20 and 22 g, were purchased from CLEA Japan, Inc. (Tokyo, Japan). Male mice were chosen, based on previous publications of pneumonectomy models. [15] The mice were kept on a 12-h light/dark cycle with free access to food and water. All experiments were conducted in accordance with protocols approved by the Animal Experimentation Committee of Keio University (Permit Number: 09076), and was performed in compliance with its Animal Experimental Guidelines. The animals were randomly assigned to groups administered a) lactated Ringer’s solution (control), b) 5% recombinant human serum albumin (rHSA), c) HbV suspension mixed with rHSA (HbV/rHSA), and d) mouse red blood cells (mRBCs) suspended in rHSA (mRBCs/rHSA) as a transfusion substitute. The hemoglobin concentrations were set at 8.6 g/dl, and the albumin concentrations were set at 5.0 g/dl (5%) in both the HbV/rHSA and mRBC/rHSA groups.[16]

### Preparation of artificial oxygen-carrying HbVs

HbVs were prepared under sterile conditions, as described previously.[6,17] Briefly, human Hb was purified from outdated donated blood provided by the Japanese Red Cross Society (Tokyo, Japan) by pasteurization and nanofiltration. Hb was then stabilized by carbonylation (HbCO) and concentrated by ultrafiltration to 38 g/dl. Subsequently, pyridoxal 5'-phosphate (PLP; Sigma Chemical Co., St. Louis, MO) was added to the HbCO solution as an allosteric effector at a PLP/Hb tetramer molar ratio of 1.0. The Hb-PLP solution was then mixed with lipids and encapsulated in vesicles. The lipid bilayer was composed of a mixture of 1,2-dipalmitoyl-sn-glycero-3-phosphatidylcholine, cholesterol, 1,5-O-dihexadecyl-N-succinyl-l-glutamate, and 1,2-distearoyl-sn-glycerol-3- phosphatidylethanolamine-N-PEG5000 (NOF Corp., Tokyo, Japan) at molar ratios of 5: 4: 0.9: 0.03, respectively. The particle diameter was regulated by extrusion through membrane filters. Encapsulated HbCO was converted to HbO_2_ by exposing the liquid membranes of HbVs to visible light under an O_2_ atmosphere. HbVs were suspended in a physiological salt solution, filter-sterilized, and deoxygenated with bubbling N_2_ prior to storage. The physicochemical parameters of the HbV were as follows: particle diameter, 252 ± 53 nm; [Hb], 10 g/dl; [lipids], 6–7 g/dl; and oxygen affinity (P_50_), 25–28 Torr (Fig 1).

For the HbV/rHSA group, the HbVs were suspended in a 5% albumin solution: the vesicle suspension was mixed with 25% rHSA solution (Nipro Corp. Osaka, Japan) at a proportion of 8.6 to 1.4. This solution was used as a colloidal resuscitative fluid containing 5% rHSA. The final HbV solution contained hemoglobin at a concentration of 8.6 g/dl. For the mRBC/rHSA group, the washed erythrocyte suspension was prepared as follows. After two donor mice were systemically heparinized with 50 U of heparin sodium/mouse, blood was withdrawn via the abdominal aorta under deep anesthesia via subcutaneous injection with ketamine (100 mg/kg) and xylazine (10 mg/kg). This blood was subsequently centrifuged (4°C; 2,000 × *g*, 15 min), and the RBCs were washed twice with physiological saline. After removing the plasma component, the RBCs were suspended in rHSA solution. The hemoglobin and rHSA concentrations were adjusted by dilution with saline to 8.6 g/dl and 5%, respectively. [16] For the rHSA group, a 5% albumin solution was prepared by diluting a 25% rHSA solution with saline.

### Isovolemic 40%-exchange transfusion

Mice were anesthetized via subcutaneous injection with ketamine (100 mg/kg) and xylazine (10 mg/kg). The mice were weighed before the introduction of a polyethylene catheter into the left carotid artery to collect blood. An estimated 40% of the total circulating blood volume of the mice was replaced by solutions of HbV suspension mixed with rHSA (HbV/rHSA), mRBCs suspended in rHSA (mRBCs/rHSA), 5% recombinant human serum albumin solution (rHSA), or lactated Ringer’s solution, which were administered via tail vein injections.[8] Based on previous rodent experiments, we estimated the systemic blood volume to be 56 ml/kg body weight.[16] The cycle of slowly removing mouse blood (0.1 ml) and replacement with an equal volume of transfusion substitute was repeated 6 times to exchange ~40% of the circulating blood volume. Samples from the first blood collection were used as baseline values. After the blood replacement, the catheter was removed and the skin was closed with a suture.

### Left surgical pneumonectomy

After the replacement of 40% of the blood volume, the mice underwent orotracheal intubation with an 18-gauge catheter and were connected to a rodent ventilator set to a rate of 100 breaths/min, 10 ml/kg tidal volume, 2 cm H_2_O positive end-expiratory pressure, and 0.21 inspired O_2_ concentration. A posterolateral incision was performed, followed by a thoracotomy at the 5^th^ intercostal space. The hilum was ligated with a 5–0 silk suture, and the lung was excised. The 5^th^ intercostal space was closed with a single suture, and the skin and muscle incisions were closed with 4 loose sutures. After removal of the left lung, the tidal volume was reduced to 7.5 ml/kg to prevent the development of ventilator-induced lung injury.[15] The duration of mechanical ventilation for all surgical procedures was set to 5 min.

### Monitoring of survival, body weight, daily food intake, and spontaneous activity

After surgery, the mice were returned to separate cages and fed a standard laboratory animal diet (Oriental Yeast Co., Ltd., Tokyo, Japan) *ad libitum*. The health of the mice was monitored every four h for the first 24 h after the surgery and every 12 h thereafter. To minimize the pain and distress of mice, carprofen was administered subcutaneously at a dose of 5 mg/kg body weight. The animals were euthanized by exsanguination via the caudal *vena cava* under deep anesthesia with subcutaneous injection of ketamine (100 mg/kg) and xylazine (10 mg/kg), when either emaciation or respiratory distress was observed. Their survival was monitored and the effects of HbV on survival were measured (n = 10). Some cages were equipped with rotating running wheels, which the mice could use *ad libitum* (n = 5). The number of daily running-wheel revolutions was counted as a measure of spontaneous activity. Changes in body weight, daily food intake, and spontaneous activity were monitored up through the 7^th^ postoperative day to ascertain the rate of postoperative recovery (n = 10 mice initially; thereafter measured with the surviving mice).[18] Daily food intake was calculated as the weight change of food in the feed tray multiplied by the number of calories per unit weight (3.59 kcal/g).

Other mice were randomly selected from each group and euthanized on the 1^st^, 3^rd^, and 7^th^ postoperative day with the injection of ketamine (100 mg/kg) and xylazine (10 mg/kg), and blood was drawn from the caudal *vena cava* (n = 10 in each group at each time point). These mice were used for a) blood cell counts, b) histopathology and immunohistochemistry, and c) serum cytokine measurements. Hematocrits were measured with glass capillaries. The major internal organs, such as the spleen, liver, and lungs, were weighed after the blood was drained on the 1^st^, 3^rd^, 7^th^, and 14^th^ postoperative days to ascertain the impact of HbV transfusions on the reticuloendothelial system, which may trap HbVs as well as old blood cells.

### Serum cytokine concentrations

We measured the serum concentrations of pro-inflammatory cytokines to examine inflammatory responses against surgical stress and transfusion. The blood samples were centrifuged at 5,000 × *g* for 10 min to separate the sera, which were stored at –80°C until analysis. The sera were then ultracentrifuged at 50,000 × *g* for 20 min to separate the HbV particles from the plasma to prevent their interference in the multi-cytokine assay. To determine the serum cytokine profiles, we used a Bio-Plex suspension array technique (Bio-Rad Laboratories, Hercules, CA), which enabled simultaneous measurements of interleukin (IL)-6, tumor necrosis factor (TNF)- α, granulocyte colony-stimulating factor (G-CSF), and interferon (IFN)- γ (n = 10).

### Histopathology and immunohistochemical staining of hypoxia-inducible factor-1 alpha (HIF-1α) protein

Organs, including the spleen, liver, kidneys, heart, lungs, and brain on the 1^st^ postoperative day were fixed in a 10% formalin neutral buffer solution, embedded in paraffin, sliced into 4-μm sections, and stained with hematoxylin and eosin for histopathologic examination. Paraffin-embedded organs were also processed for immunohistochemical staining, using an anti HIF-1α antibody (Millipore Corp., Billerica, MA) to study the hypoxic conditions of pneumonectomized mice.[19]

### Statistical analysis

The data are presented as the mean ± standard deviation. Changes from baseline were compared using Student’s *t*-test. Differences between the HbV/rHSA or mRBCs/rHSA group vs. the rHSA group at each time point were compared using one-way analysis of variance and Dunnett’s test. Kaplan—Meier survival curves associated with each transfusion substitute were constructed. Food intake, body weight, and daily activity were measured and compared by repeated-measures analysis of variance. When significant differences were detected, *post-hoc* analyses were performed using Bonferroni’s corrections. A *P* value < 0.05 was considered statistically significant. The data were analyzed with StatView-J software, version 5.0 (Abacus Concepts Inc., Berkeley, CA).

## Results

### Survival after 40% blood exchange and pneumonectomy

**Fig 2:**
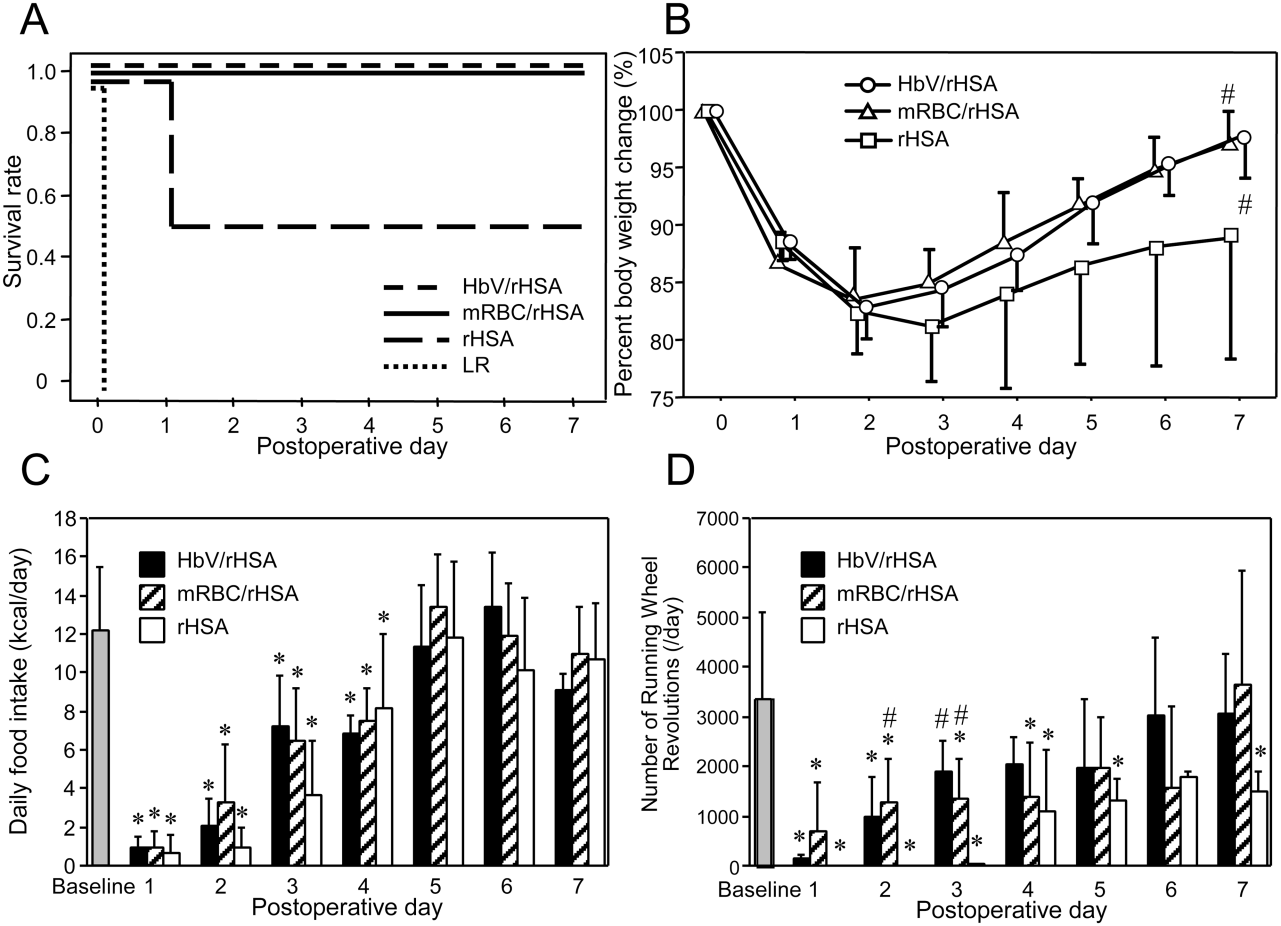
Differences in (A) survival rate, n = 10, (B) percent change in body weight (n = 10 initially in each group, n = 5 after the 1^st^ postoperative day in the rHSA group), (C) daily food intake (n = 10 initially each group, n = 5 after the 1^st^ postoperative day in the rHSA group), and (D) daily number of running wheel revolutions (n = 5 between the baseline period and the 7^th^ postoperative day [POD] in the indicated study groups). Values are shown as the mean ± SD. *P < 0.05 versus baseline (Student’s t-test), #*P* < 0.05 versus the rHSA group (Dunnett’s test).

All mice that underwent 40% blood exchange with HbV/rHSA or mRBCs/rHSA before left pneumonectomy survived for ≥7 days thereafter. In contrast, all mice in the control group died within a few h (1.4 ± 0.6 h). In the rHSA group, 5 of 10 mice died within 24 h (10 ± 4 h) and the remaining mice survived ≥7 days (Fig 2A). We used humane endpoints, and all mice suffering from either emaciation or respiratory distress were euthanized by exsanguination under deep anesthesia. No mice died unexpectedly.

### Postoperative weight loss and regain

Changes in body weight were compared among the HbV/rHSA, mRBC/rHSA, and rHSA-only groups, as no mice in the control group survived (Fig 2B). In the HbV/rHSA and mRBC/rHSA groups, the greatest body weight loss occurred on the 2^nd^ postoperative day, followed by a rapid weight regain. In the rHSA group, the 5 surviving mice incurred the greatest loss in body weight on the 3^rd^ postoperative day and tended to remain lighter than the mice in the HbV/rHSA and mRBC/rHSA groups throughout the study period, although no significant differences were observed. Significant differences were found between the HbV/rHSA and rHSA groups and between the mRBC/rHSA and rHSA groups (*P* < 0.05 for both comparisons) on the 7^th^ postoperative day.

### Postoperative food intake

Comparisons of postoperative daily food intake among the 3 groups of surviving mice after 40% blood exchange and pneumonectomy are shown in Fig 2C. Compared with food intake before surgery, the amount of food consumption decreased sharply on the 1^st^ postoperative day. Subsequently, food intake increased daily and similarly thereafter in the 3 experimental groups. No significant differences were observed between the groups throughout the study period.

### Postoperative daily spontaneous activity

Spontaneous activity among the groups of surviving mice was compared (Fig 2D). In the HbV/rHSA and mRBC/rHSA groups, the mice began to crawl in their cages and drink water within a few h after surgery, whereas the physical activity of survivors in the rHSA group was markedly limited for 3 days. The number of postoperative running wheel revolutions in the rHSA group was considerably below the approximate number of 3,000 revolutions/day measured preoperatively. The absence of running activity persisted longer in the rHSA group than in the HbV/rHSA and mRBC/rHSA groups. Significant differences (*P* < 0.05) were also observed between the mRBC/rHSA and rHSA groups on the 2^nd^ and 3^rd^ postoperative days, as well as between the HbV/rHSA and rHSA groups on the 3^rd^ postoperative day (*P* < 0.05). Spontaneous activity measured in the HbV/rHSA and mRBC/rHSA groups was significantly greater than in the rHSA group (both comparisons *P* < 0.01) throughout the study period, whereas no significant difference was found between the HbV/rHSA and mRBC/rHSA groups.

### Hematological changes

**Fig 3:**
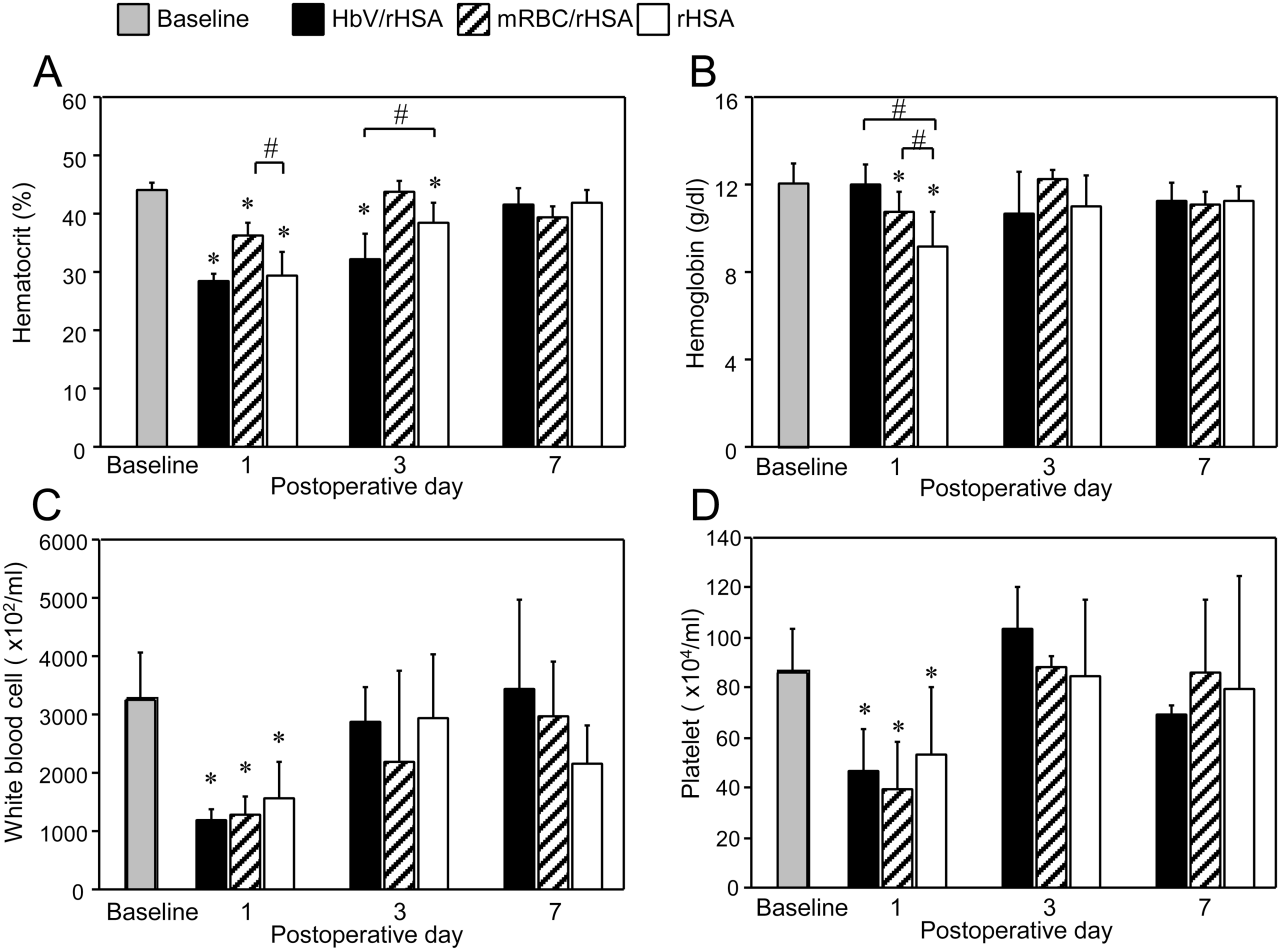
Changes in (A) hematocrit levels, (B) hemoglobin concentrations, (C) white blood cell counts, and (D) platelet counts between the baseline period and the 7^th^ postoperative day (POD) in the indicated study groups. Values are shown as the mean ± SD. *n* = 10 in each group. *P < 0.05 versus baseline (Student’s t-test), #*P* < 0.05 versus the rHSA group (Dunnett’s test).

The mean hematocrit was approximately 45% before the exchange transfusions (Fig 3A). On the 1^st^ postoperative day, it fell to 27% in the HbV/rHSA and rHSA groups and to 36% in the mRBC/rHSA group. In the HbV/rHSA group, for which hemoglobin was measured with a hematology analyzer using the same settings as for RBCs, the decrease in hemoglobin concentration on the 1^st^ postoperative day was not significant, whereas it was significant in the rHSA group (HbV/rHSA vs. rHSA; *P* < 0.05, Fig 3B). On the 3^rd^ postoperative day, the hematocrits in the rHSA and the HbV/rHSA groups were significantly different (*P* < 0.01). On the 7^th^ postoperative day, the hematocrit increased to 40% in the HbV/rHSA and rHSA groups. The white blood cells and platelets fell to 50% of baseline on the 1^st^ postoperative day in the 3 groups, and increased thereafter (Fig 3C and 3D).

### Changes in splenic weight

**Fig 4:**
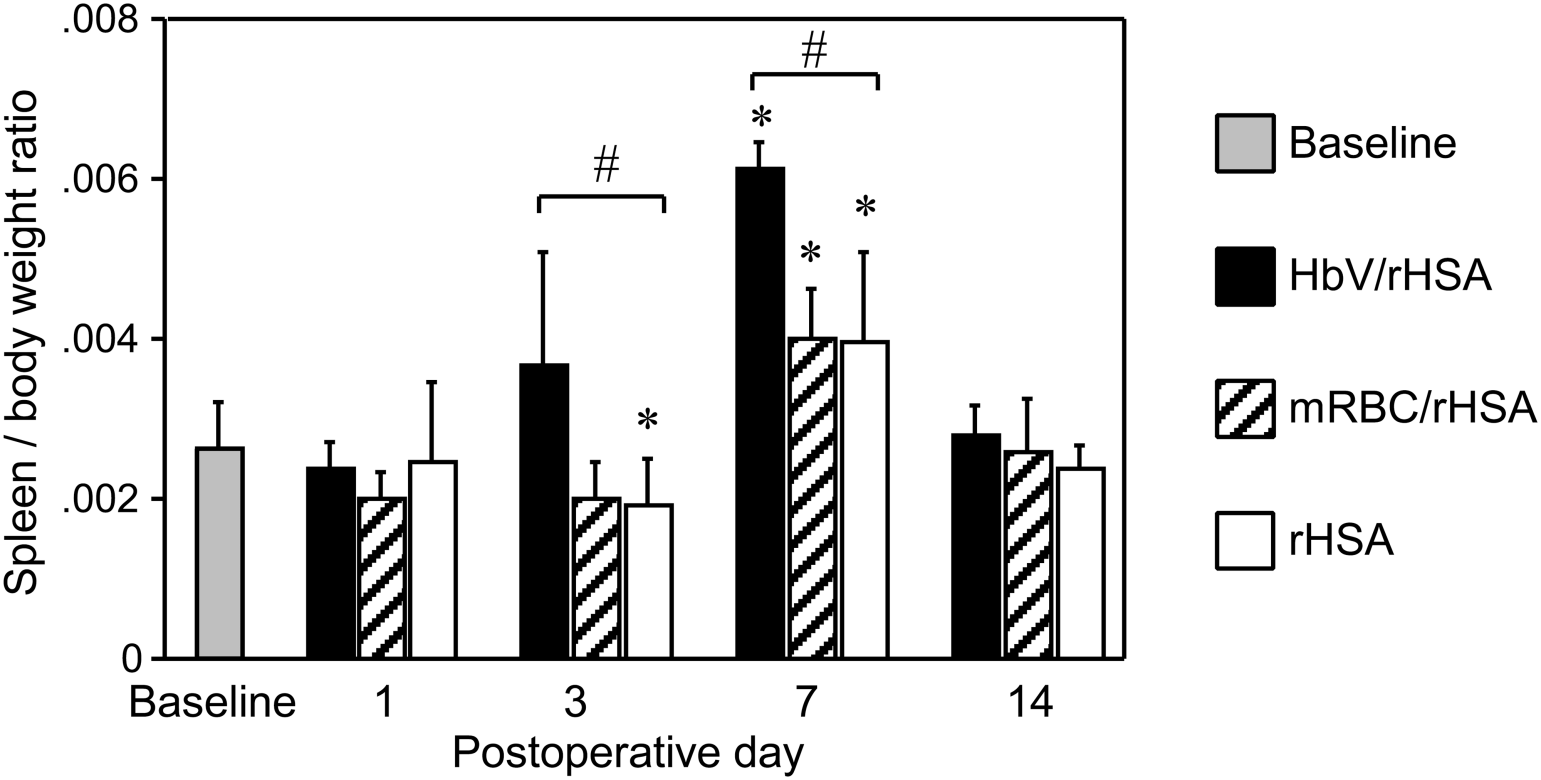
Changes in spleen/body weight ratio between the baseline period and the 14^th^ postoperative day (POD) in the indicated study groups. Values are shown as the mean ± SD. *n* = 10 in each group. *P < 0.05 versus baseline (Student’s t-test), #*P* < 0.05 versus the rHSA group (Dunnett’s test).

Compared with baseline weights, splenic weights more than doubled in the HbV/rHSA group by the 7^th^ postoperative day (*P* < 0.001) and returned to baseline by the 14^th^ postoperative day (Fig 4). Splenic weights also increased significantly compared with baseline weights in the mRBC/rHSA (*P* < 0.005) and rHSA (*P* < 0.05) groups on the 7^th^ postoperative day, and then returned to baseline. The spleen was significantly heavier in the HbV/rHSA group than in the rHSA groups on the 3^rd^ and the 7^th^ postoperative days (*P* < 0.05 for both comparisons). No significant weight changes in the livers and lungs were observed between groups.

### Inflammatory cytokine responses

**Fig 5:**
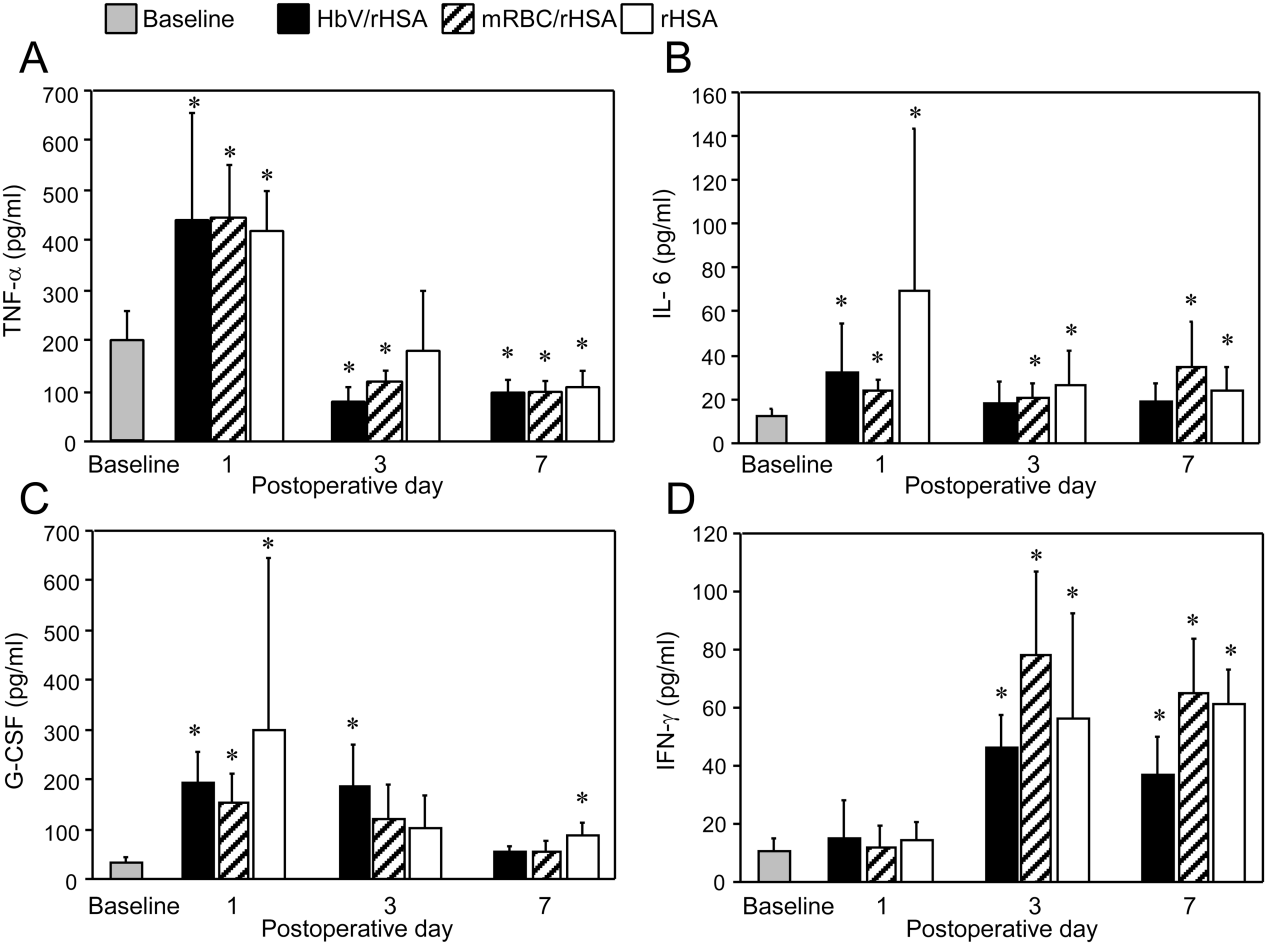
Changes in serum concentrations of (A) TNF-α, (B) IL-6, (C) G-CSF, and (D) IFN-γ between the baseline period and the 7^th^ postoperative day (POD), in the indicated study groups. Values are shown as the mean ± SD. n = 10 in each group. *P < 0.05 versus baseline (Student’s t-test).

The serum concentrations of TNF-α, IL-6, and G-CSF were significantly higher on the 1^st^ postoperative day than at baseline in the 3 groups, and decreased thereafter in each group (Fig 5). The IFN- γ concentration was low on the 1^st^ postoperative day and rose on the 3^rd^ and 7^th^ postoperative days. The serum concentrations of TNF- α, IL-6, G-CSF, and IFN-γ were similar among the 3 groups at each time point.

### Histopathology of the major organs

**Fig 6:**
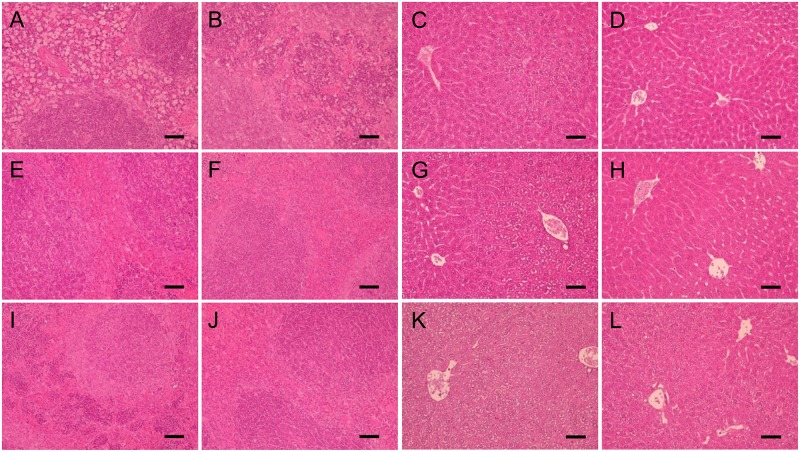
Representative histological sections of mouse spleens on the 1^st^ (A, E, I) and 7^th^ (B, F, J) postoperative days, and livers on the 1^st^ (C, G, K) and 7^th^ (D, H, K) postoperative days after transfusion with HbV/rHSA (A, B, C, D), mRBCs/rHSA (E, F, G, H) or rHSA (I, J, K, L). Bars = 50 μm. Hematoxylin/eosin stain.

Histopathological examinations of the spleens on the 1^st^ and 7^th^ days after 40% blood exchange and pneumonectomy in the HbV, mRBC/rHSA, and rHSA groups are shown in Fig 6. The splenic red pulp zones in the HbV/rHSA group were filled with a large quantity of HbV/rHSA particles on the 1^st^ postoperative day (Fig 6A), with macrophages apparently entrapping the HbVs. The accumulation of HbVs was obscure and a prominent nest formation of erythroblasts was present in the splenic cord on the 7^th^ postoperative day in the HbV/rHSA group, indicating the occurrence of extramedullar erythropoiesis (Fig 6B). A prominent nest formation was also present in the spleens of mice in the rHSA group on the 1^st^ and the 7^th^ postoperative days (Fig 6I and 6J), whereas the spleens of the mRBC/rHSA group were mostly unchanged on both the 1^st^ and the 7^th^ postoperative days (Fig 6E and 6F).

In the rHSA group a prominent and diffuse cytoplasmic vacuolation of the hepatocytes was noted (Fig 6K), indicating severe ischemia, although mild cytoplasmic vacuolation was limited around the central veins in the HbV/rHSA and mRBC/rHSA groups (Fig 6C and 6G) on the 1^st^ postoperative day. Nuclear vacuolation of the hepatocytes was also prominent in the rHSA group on the 1^st^ postoperative day. The cytoplasmic and nuclear vacuolation lasted up to the 7^th^ postoperative day in the livers of the rHSA group (Fig 6L), whereas, in the HbV/rHSA and mRBC/rHSA groups, the hepatocytes had normalized by the 7^th^ postoperative day (Fig 6D and 6H).

**Fig 7:**
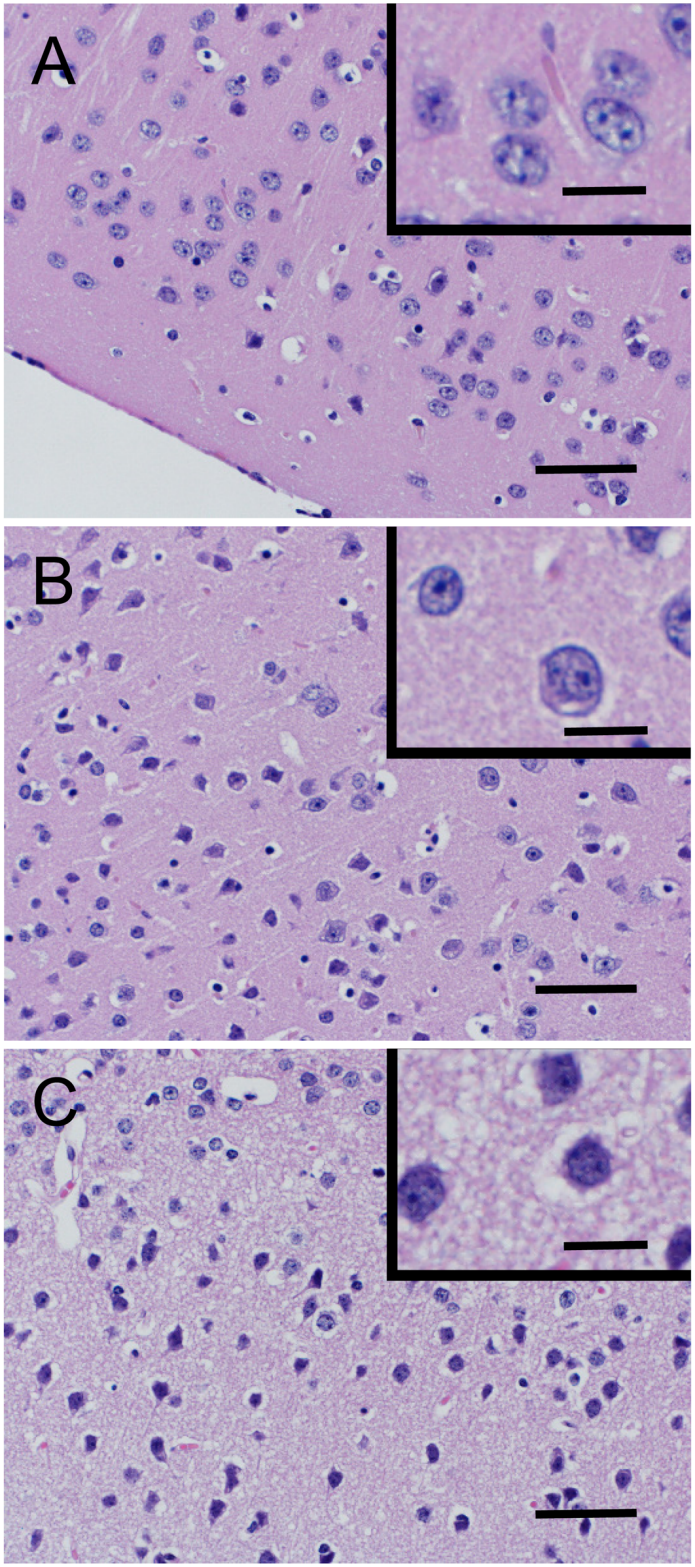
Representative histological sections of mouse cerebral cortex on the 1^st^ postoperative days after transfusion with HbV/rHSA (A), mRBC/rHSA (B), or rHSA (C). Bars = 50 μm. Inset bars = 10 μm. Hematoxylin/eosin stain.

Furthermore, cerebral cortex sections of the rHSA group demonstrated chromatin condensation and cytoplasmic vacuolization associated with neural cell degeneration were prominent on the 1^st^ postoperative day (Fig 7A), while these phenomena were obscure in the HbV/rHSA and mRBC/rHSA groups (Fig 7B and 7C).

No significant changes were observed in the kidneys, heart, and lungs throughout the study period among the 3 groups (not shown).

### Immunohistochemistry of HIF-1α protein

**Fig 8:**
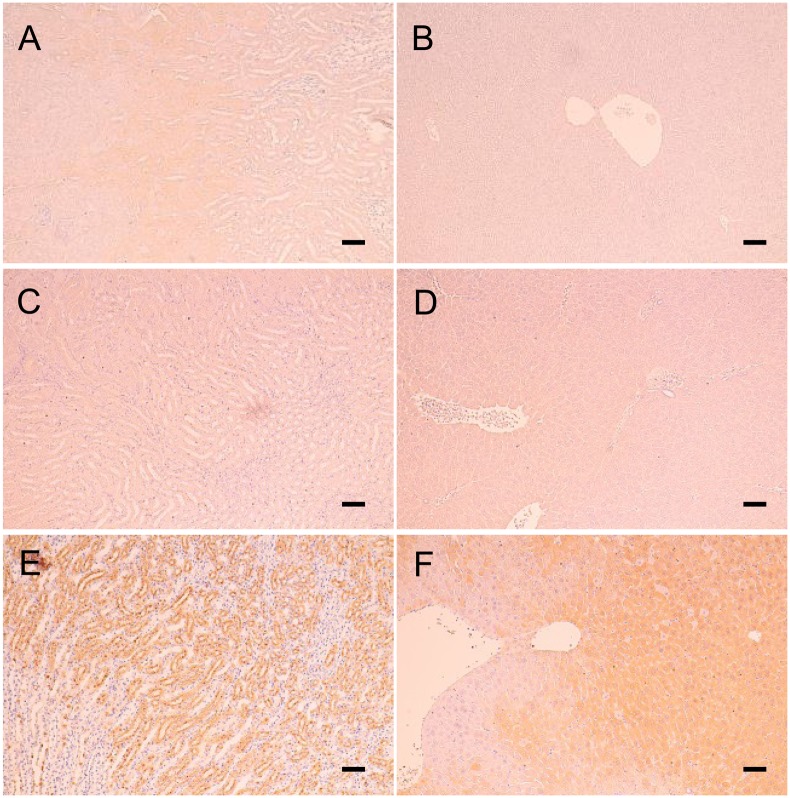
Immunohistochemical staining for HIF-1α in mouse kidneys (A, C, E) and livers (B, D, F) on day 1 after transfusion with HbV/rHSA (A, B), mRBCs/rHSA (C, D), or rHSA (E, F). Bars = 50 μm.

HIF-1α staining in the proximal renal tubules of the rHSA group was prominent (Fig 8E), whereas it was weak in the HbV/rHSA and mRBC/rHSA groups (Fig 8A and 8C). The hepatocytes were diffusely stained by the anti-HIF-1α antibody in the rHSA group (Fig 8F), while no HIF-1α staining was observed in the HbV/rHSA and mRBC/rHSA groups (Fig 8B and 8D). No significant change in HIF-1α expression was observed in the spleen, heart, and lungs.

## Discussion

The infusion of HbVs suspended in serum albumin compensated for a 40% blood loss in pneumonectomized mice with severely impaired lung functions and did not interfere with their recovery from surgical stress. These hemoglobin vesicles, which can be preserved for years without risk of infection and administered to patients with all blood types, might be a welcome substitute for the perioperative transfusion of RBCs.

In preliminary experiments, mice that underwent 40%-exchange transfusion with lactated Ringer’s solution or rHSA without pneumonectomy survived over the course of the study period. However, all mice that underwent 40%-exchange transfusion with lactated Ringer’s solution followed by pneumonectomy died within a few h, and 50% of the mice that underwent 40%-exchange transfusion of rHSA followed by pneumonectomy died within 24 h. Ringer’s solution is a crystalloid osmotic agent that fails to maintain an effective circulating blood volume, whereas albumin solution provides colloid osmotic pressure, which maintains plasma in the blood vessels and helps to improve blood circulation. These properties resulted in differences in survival rates. By contrast, mice that underwent exchange infusion with HbV/rHSA showed survival similar to mice transfused with mRBC/rHSA, suggesting that HbV served the same function as mRBCs, even in the context of depressed pulmonary function following pneumonectomy.

The immediate postoperative loss and subsequent regain of body weight were similar in the HbV/rHSA and mRBC/rHSA groups, while the recovery of body weight in the rHSA group tended to be delayed for 7 days. The daily food intake of the HbV/rHSA and mRBC/rHSA groups increased similarly, while that of the rHSA group was limited for the first 3 days. The surgical stress of anesthesia, pneumonectomy, and blood dilution created a catabolic state, while dehydration and decreased food intake caused weight loss. HbV/rHSA administration appeared to protect the metabolic system, based on the measured increases in food consumption and body weight recovery, which were similar to those observed in the mRBC/rHSA group. In addition, spontaneous activity in the HbV/rHSA and mRBC/rHSA groups increased similarly, while that of the rHSA group remained low for the first 3 days. However, it has been reported that exercise with running wheels influences weight changes, food intake, and the expression of inflammatory markers in mice. [20] In this study, body weight changes, food intake, and cytokine measurements were evaluated in 10 mice, but 5 out of 10 mice in the HbV/rHSA and mRBC/rHSA groups did not have access to the running wheels, while all 5 surviving mice in the rHSA group had access to the wheels. If all mice in the HbV/rHSA and mRBC/rHSA groups had access to the wheels, the body weight changes might have decreased and the food intake might have increased in both groups, which in turn could have influenced differences from the rHSA group.

Hematopoiesis did not appear to be impaired by HbV administration, as the decrease and recovery of the hematocrit was similar in the HbV/rHSA and rHSA groups. HbV is methoxylated and loses its oxygen-carrying capacity in a few days in vivo.[21] Our results, however, showed that the function of HbV was durable enough to support the mice during the critical phase after significant acute normovolemic hemodilution and pneumonectomy.

Data from our previous studies of the biokinetics of radiolabeled HbV showed that it entered the reticuloendothelial system.[12,22] HbV was entrapped in the splenic red pulp and caused splenomegaly, which disappeared within 14 days after administration in this study and in our previous studies.[21] However, splenomegaly was prominent in the HbV/rHSA, mRBC/rHSA, and rHSA groups on the 7^th^ postoperative day. In our previous experiment, splenomegaly was observed on the 7^th^ day after 40% exchange-transfusion with rat RBC/rHSA solution in a rat model, which was associated with hemosiderin deposition in the spleen.[16] This finding is compatible with the present results. The washed RBCs might show decreased deformability and be as fragile as old RBCs, entrapped in the spleen, and then iron originating from heme could be deposited in the spleen.[16] The cause of splenomegaly in the rHSA group might be the manifestation of extramedullary erythropoiesis as a nest formation. This possibility is compatible with our previous findings that rats exposed to anemic conditions showed enhanced extramedullary erythropoiesis in the spleen, which was associated with increased erythropoietin secretion from the kidney. [16] Prolonged splenomegaly in the HbV/rHSA group might also be explained by extramedullar erythropoiesis.[10,21] We previously reported that the harmful release of porphyrin-degradation products such as heme iron or bilirubin into blood did not occur after HbV entrapment in the spleen, suggesting that heme iron was physiologically excreted as bilirubin in the bile.[16]

Histopathologic ischemic changes in the liver were mitigated by HbV during the acute phase after blood loss and pneumonectomy, and recovery occurred within 7 days after the operation, as observed in the mRBC/rHSA group. In addition, administration of HbV/rHSA solution or mRBC/rHSA solution mitigated hypoxic damage in the cerebral cortex, which consumes a high amount of oxygen and is particularly sensitive to hypoxic conditions. Immunohistochemical staining for HIF-1α also suggested that the hepatocytes and kidney tubules in the HbV/rHSA group received the same protection against hypoxia as did the mRBC/rHSA group, in contrast to the HIF-1α prominent expression observed in the rHSA group. Because HiF-1α is a biologic sensor of hypoxia, these observations indicated that HbV provides oxygen to the tissues of major hypoxia-sensitive organs under conditions of depressed lung function after pneumonectomy.[19]

Exchange transfusion with HbV caused no adverse inflammatory responses in pneumonectomized mice.[21] TNF-α, a major pro-inflammatory cytokine, was involved in the early-phase cytokine response after pneumonectomy; however, the TNF-α level decreased within 3 days despite transfusion with HbV/rHSA, mRBC/rHSA, or rHSA. The level of IL-6, another early-phase cytokine, also increased on the 1^st^ postoperative day before decreasing similarly in the HbV/rHSA and rHSA groups. G-CSF changed similarly over time. The level of the late-phase cytokine, IFN-γ, was elevated similarly on the 3^rd^ postoperative day in the HbV/rHSA, mRBC/rHSA, and rHSA groups. However, as discussed above, the differences of cytokine levels between the HbV/rHSA vs. rHSA groups, and mRBC/rHSA vs. rHSA groups might have been influenced by the different levels of access to the exercise wheels.[20]

Our study has some limitations with respect to extrapolating to a human clinical setting. Because no production facility has been established to generate HbVs in large quantities, many HbVs could not be used at a time. Therefore, we used small animals. Evaluating HbVs in bigger animals such as dogs will be necessary. In addition, to prioritize the survival of small animals, we abandoned serial measurements of hemodynamics and blood oxygenation. We did not perform quantification of HIF-1α expression, which is used as a marker to evaluate the oxygenating effects of HbVs on hypoxic organs, because we examined the effects by immunohistochemistry. Finally, the current study lacked an evaluation of wound healing, including the measurement of collagen synthesis in wounds and the tensile strengths of suture lines.

In conclusion, acute normovolemic hemodilution with HbV, an artificial oxygen carrier, suspended in rHSA, in which 40% of estimated total blood volume was exchanged, did not adversely affect recovery after pneumonectomy in mice, thereby avoiding organ hypoxia and the need for RBC transfusion. HbVs might be clinically applicable for treating perioperative blood loss as an alternative for RBC transfusion.

## References

[1] Ministry of Health, Labour and Welfare, Japan. Proceedings of Blood Donation Promotion Committee, Pharmaceutical Affairs and Food Sanitation Council on 2 December, 2014. < http://www.mhlw.go.jp/file/05-Shingikai-11121000-Iyakushokuhinkyoku-Soumuka/0000067177.pdf>, (2014) (Last accessed 08/25/2016).

[2] Chang TM. Blood substitutes based on modified hemoglobin prepared by encapsulation or crosslinking: an overview. Biomater Artif Cells Immobilization Biotechnol. 1992;20:159–179. 10.3109/10731199209119634 1391433

[3] Winslow RM. Blood substitutes—a moving target. Nat Med 1995;1:1212–1215. 7584998 10.1038/nm1195-1212

[4] Tsuchida E. Introduction: Overview and Perspective Artificial Red Cells. Tsuchida E., Ed. New York: Wiley;1995:1–20.

[5] Sakai H, Hamada K, Takeoka S, Nishide H, Tsuchida E. Physical properties of hemoglobin vesicles as red cell substitutes. Biotechnol Prog. 1996;12:119–125. doi: 10.1021/bp950068w 8845102 10.1021/bp950068w

[6] Sakai H, Hara H, Yuasa M, Tsai AG, Takeoka S, Tsuchida E et al Molecular dimensions of Hb-based O(2) carriers determine constriction of resistance arteries and hypertension. Am J Physiol Heart Circ Physiol. 2000;279:H908–H915. 10993749 10.1152/ajpheart.2000.279.3.H908

[7] Izumi Y, Sakai H, Kose T, Hamada K, Takeoka S, Yoshizu A et al Evaluation of the capabilities of a hemoglobin vesicle as an artificial oxygen carrier in a rat exchange transfusion model. ASAIO J. 1997;43:289–297. 9242942

[8] Sakai H, Masada Y, Horinouchi H, Yamamoto M, Ikeda E, Takeoka S et al Hemoglobin-vesicles suspended in recombinant human serum albumin for resuscitation from hemorrhagic shock in anesthetized rats. Crit Care Med. 2004;32:539–545. doi: 10.1097/01.CCM.0000109774.99665.22 14758176 10.1097/01.CCM.0000109774.99665.22

[9] Seishi Y, Horinouchi H, Sakai H, Kobayashi K. Effect of the cellular-type artificial oxygen carrier hemoglobin vesicle as a resuscitative fluid for prehospital treatment: experiments in a rat uncontrolled hemorrhagic shock model. Shock. 2012;38:153–158. doi: 10.1097/SHK.0b013e31825ad7cf 22777109 10.1097/SHK.0b013e31825ad7cf

[10] Sakai H, Horinouchi H, Tomiyama K, Ikeda E, Takeoka S, Kobayashi K et al Hemoglobin-vesicles as oxygen carriers: influence on phagocytic activity and histopathological changes in reticuloendothelial system. Am J Pathol. 2001;159:1079–1088. doi: 10.1016/S0002-9440(10)61783-X 11549600 10.1016/S0002-9440(10)61783-X PMC1850444

[11] Sakai H, Horinouchi H, Tsuchida E, Kobayashi K. Hemoglobin vesicles and red blood cells as carriers of carbon monoxide prior to oxygen for resuscitation after hemorrhagic shock in a rat model. Shock. 2009;31:507–514. doi: 10.1097/SHK.0b013e318188f83d 18827742 10.1097/SHK.0b013e318188f83d

[12] Taguchi K, Maruyama T, Iwao Y, Sakai H, Kobayashi K, Horinouchi H et al Pharmacokinetics of single and repeated injection of hemoglobin-vesicles in hemorrhagic shock rat model. J Control Release. 2009;136:232–239. doi: 10.1016/j.jconrel.2009.02.009 19245823 10.1016/j.jconrel.2009.02.009

[13] Yamamoto M, Horinouchi H, Kobayashi K, Seishi Y, Sato N, Itoh M et al Fluid resuscitation of hemorrhagic shock with hemoglobin vesicles in Beagle dogs: pilot study. Artif Cells Blood Substit Immobil Biotechnol. 2012;40:179–195. doi: 10.3109/10731199.2011.637929 22288842 10.3109/10731199.2011.637929

[14] Yamazaki M, Aeba R, Yozu R, Kobayashi K. Use of hemoglobin vesicles during cardiopulmonary bypass priming prevents neurocognitive decline in rats. Circulation. 2006;114:I220–I225. doi: 10.1161/CIRCULATIONAHA.105.000562 16820576 10.1161/CIRCULATIONAHA.105.000562

[15] Tajima A, Kohno M, Watanabe M, Izumi Y, Tasaka S, Maruyama I et al Occult injury in the residual lung after pneumonectomy in mice. Interact Cardiovasc Thorac Surg. 2008;7:1114–1120. doi: 10.1510/icvts.2007.170456 18713778 10.1510/icvts.2007.170456

[16] Sakai H, Horinouchi H, Yamamoto M, Ikeda E, Takeoka S, Takaori M et al Acute 40 percent exchange-transfusion with hemoglobin-vesicles (HbV) suspended in recombinant human serum albumin solution: degradation of HbV and erythropoiesis in a rat spleen for 2 weeks. Transfusion. 2006;46:339–347 doi: 10.1111/j.1537-2995.2006.00727.x 16533274 10.1111/j.1537-2995.2006.00727.x

[17] Sakai H, Tomiyama K, Masada Y, Takeoka S, Horinouchi H, Kobayashi K et al Poly(ethylene glycol)-conjugation and deoxygenation enable long-term preservation of hemoglobin-vesicles as oxygen carriers in a liquid state. Bioconjug Chem. 2000;11:425–432. 10821660 10.1021/bc990173h

[18] Kohno M, Watanabe M, Izumi Y, Tasaka S, Kitagawa Y, Maruyama I et al Mitigation of occult lung injury by pneumonectomy via minithoracotomy in mice. Thorac Cardiovasc Surg. 2012;60:124–130. doi: 10.1055/s-0030-1271011 21544787 10.1055/s-0030-1271011

[19] Nogami Y, Kinoshita M, Takase B, Ogata Y, Saitoh D, Kikuchi M et al Liposome-encapsulated hemoglobin transfusion rescues rats undergoing progressive hemodilution from lethal organ hypoxia without scavenging nitric oxide. Ann Surg. 2008;248:310–319. doi: 10.1097/SLA.0b013e3181820c80 18650643 10.1097/SLA.0b013e3181820c80

[20] Bradley RL, Jeon JY, Liu FF, Maratos-Flier E. Voluntary exercise improves insulin sensitivity and adipose tissue inflammation in diet-induced obese mice. Am J Physiol Endocrinol Metab. 2008;295:E586–E594. doi: 10.1152/ajpendo.00309.2007 18577694 10.1152/ajpendo.00309.2007 PMC2536732

[21] Sakai H, Horinouchi H, Masada Y, Takeoka S, Ikeda E, Takaori M et al Metabolism of hemoglobin-vesicles (artificial oxygen carriers) and their influence on organ functions in a rat model. Biomaterials. 2004;25:4317–4325. doi: 10.1016/j.biomaterials.2003.11.005 15046922 10.1016/j.biomaterials.2003.11.005

[22] Taguchi K, Urata Y, Anraku M, Watanabe H, Kadowaki D, Sakai H et al Pharmacokinetic study of enclosed hemoglobin and outer lipid component after the administration of hemoglobin vesicles as an artificial oxygen carrier. Drug Metab Dispos. 2009;37:1456–1463. doi: 10.1124/dmd.109.027094 19364827 10.1124/dmd.109.027094

